# Current hospital policies on breastfeeding: a survey from Italy

**DOI:** 10.1186/s13052-024-01581-5

**Published:** 2024-01-25

**Authors:** Riccardo Davanzo, Laura Travan, Maria Lorella Giannì, Giuseppe Giordano, Silvia Perugi, Mariella Baldassarre, Antonella Soldi, Lorenzo Colombo, Isabella Mondello, Michela Pandullo, Alessia Ferrara, Elena Scarpato, Guglielmo Salvatori

**Affiliations:** 1 Institute for Maternal and Child Health, IRCCS “Burlo Garofolo”, Via dell’Istria 65/1, 34100 Trieste, Italy; 2 Fondazione IRCCS Ca’ Granda Ospedale Maggiore Policlinico, NICU, Milan, Italy; 3 Department of Clinical Sciences and Community Health, University of Milan, Milan, Italy; 4 Division of Neonatology and NICU, Azienda Ospedaliera Ospedali Riuniti Villa Sofia-Cervello, Palermo, Italy; 5 Division of Neonatology, Careggi University Hospital, Florence, Italy; 6 Department of Interdisciplinary Medicine-Neonatology and NICU, University Aldo Moro, Bari, Italy; 7 Division of Neonatology and NICU, Department of Public Health and Pediatric Sciences, Sant’Anna Hospital, University of Turin, AOU Città della Salute e della Scienza, Turin, Italy; 8 NICU, Grande Ospedale Metropolitano Bianchi-Melacrino-Morelli, Reggio Calabria, Italy; 9 Università degli Studi di Udine, Udine, Italy; 10 Department of Medical Surgical and Health Sciences, University of Trieste, Università degli Studi di Trieste Dipartimento Universitario Clinico di Scienze Mediche e Chirurgiche e della Salute, Trieste, Italy; 11 Department of Translational Medical Sciences - Section of Pediatrics, University Federico II, Naples, Italy; 12 Neonatal Intensive Care Unit, Bambino Gesù Children’s Hospital, IRCCS, 00165 Rome, Italy

**Keywords:** Breastfeeding, Policy, Hospital, Survey, Baby-Friendly Hospital Initiative

## Abstract

**Background:**

The availability of an appropriate newborn feeding policy is an essential component of the promotion of breastfeeding in health facilities. The Italian Society of Neonatology (SIN) and the Italian Society of Paediatrics (SIP) have run an online survey among Maternity Hospitals to explore the existing breastfeeding policies and their characteristics.

**Methods:**

Between February and April 2023, an online survey was carried out among 110 Italian maternity hospitals with a Neonatal Intensive Care Unit (NICU).

**Results:**

Forty-nine Maternity Hospitals completed the online questionnaire. Twenty out of 49 (40.8%) reported to have a breastfeeding policy. When a policy is available, its quality appears to be suboptimal because of lack of inclusion of a family representative in the policy working group, limited options for translating breastfeeding policy into minority languages, lack of periodic assessment of their implementation.

**Conclusion:**

Currently, only a limited number of Italian Maternity Hospitals have developed a breastfeeding policy. Additional efforts are needed for their improvement as well as implementation.

**Supplementary Information:**

The online version contains supplementary material available at 10.1186/s13052-024-01581-5.

## Background

Breastfeeding is beneficial in providing individualized nutrition with lifelong effects to the nursed infant and in improving maternal health, particularly by reducing the risk of female reproductive cancers and empowering new mothers [1] Despite this, breastfeeding rates in high-income countries are usually low (equal to 18% by 6 months of age for exclusive breastfeeding) and represent a cause for concern [2].

Although breastfeeding success has multiple determinants (e.g.: cultural, educational, psychological, social, clinical, etc.), scientific evidence has demonstrated that many hospital practices at childbirth, such as skin-to-skin contact, initiation of breastfeeding within an hour of birth, rooming-in of mother and newborn, and limiting the supplementation of formula milk to breastfed infants, play a key role in facilitating both initiation and duration of breastfeeding [1].

Recognizing the essential role of Maternity hospitals, in 1991 the United Nations International Children’s Emergency Fund (UNICEF) and the World Health Organization (WHO) launched the Baby-Friendly Hospital Initiative (BFHI) in order to assist health facilities worldwide to better protect, promote and support breastfeeding [3]. The BFHI require the implementation of the Ten Steps, as a package of recommended perinatal practices aimed to increase the exclusive breastfeeding rate at discharge from Maternity Hospital and also the subsequent duration of breastfeeding. However, despite the multi-decade commitment of UNICEF and WHO at a global level and the strong efforts of the National BFH Committees in many Countries, in 2017 only 10% of infants worldwide were born in a “Baby-friendly” hospital [4].

An effective, written newborn feeding policy, routinely communicated to hospital staff and parents, is an essential component of Step 1 of the BFHI, as highlighted during the COVID-19 pandemic by the disruption of breastfeeding in Maternity Hospitals lacking an ad hoc policy [5].

Preliminarily to a nationwide hospital-based project aimed to promote breastfeeding from May 2023 to February 2025, the Italian Society of Neonatology (SIN) and the Italian Society of Paediatrics (SIP) have conducted a Survey to assess existing breastfeeding policies and their characteristics in Italian Maternity Hospitals.

## Methods

A survey was conducted through SurveyMonkey from February to April 2023. Maternity Hospitals with a Neonatal Intensive Care Uni (NICU) were identified as the target population of the Survey. The Directors of 110 Italian NICUs were invited via email to participate in the study and to complete a questionnaire comprising 16 closed-ended questions (see Supplementary Materials) developed by the Task Force on Breastfeeding of the Italian Society of Neonatology (Com.A.SIN).

The questionnaire aimed to assess the availability and key features of a breastfeeding policy, as outlined by the Academy of Breastfeeding Medicine [6]. Specifically, the questionnaire was designed to gather main information on: (a) the composition of the working group that devided the policy, (b) the inclusion in the policy of a statement on the role of health workers and the hospital general manager in protecting, promoting and supporting breastfeeding, (c) the way the policy was disseminated, (d) the need for training health professionals on breastfeeding, and (e) the guideline for prescribing milk formula upon discharge from hospital.

Completing the questionnaire took approximately 10 min.

A frequency analysis was performed on the collected data. Since not all questions in the questionnaire might have been answered by the reference professionals of the Maternity Hospitals, the percentage for each response was calculated based on the actual number of responses received.

## Results

Forty-nine out of 110 Maternity Hospitals from 15 out of 20 Italian Regions completed the online questionnaire yielding a response rate of 44.5%. Some hospitals that participated in the survey did not respond to all of the questions included in the questionnaire.

Twenty out of 49 Maternity Hospitals (40.8%) reported having a breastfeeding policy. Among these, 55% (11/20) declared that their policy was fully compliant with the International Code of Marketing of Breast-Milk Substitutes (referred to as “Code”) [7] (Table 1). 25% of these hospitals developed their policy after 2019.

**Table 1:** Development of a hospital policy on breastfeeding according to the application of the WHO International Code of Marketing of Breast-Milk Substitutes. Data from all Maternity Hospitals

Answer options	Number of hospital	Percentage
• Yes, our hospital is Baby Friendly and our Policy supports the full application of the WHO International Code of Marketing of Breast-Milk Substitutes	2/49	4.1%
• Yes, our hospital is on the way to become a Baby Friendly Hospital and our Policy supports the full application of the WHO International Code of Marketing of Breast-Milk Substitutes Code of Marketing of Breast-Milk Substitutes	9/49	18.4%
• Yes, our hospital has a Policy, that supports a partial application of the WHO International Code of Marketing of Breast-Milk Substitutes	9/49	18.4%
• No, our hospital has no Policy on Breastfeeding, although we plan to develop one	20/49	40.7%
• No, our hospital has no Policy on Breastfeeding	9/49	18.4%

In 93.3% of the hospitals, the policy is communicated to all staff members involved in mother and infant care (Table 2). In addition, the vast majority (80.0%) also display the policy in the facilities that provide health care to pregnant women, new mothers and families. In 26.7% of the hospitals the policy was made available also in the main languages spoken by the population referring to the Maternity Hospital (Table 3).

**Table 2:** Main information regarding policies on breastfeeding. Data from Maternity Hospitals with a policy

	Number of hospitals	Percentage
• The policy has been available since 2020	4/16	25.0%
• The policy has been developed by a multidisciplinary group	15/16	93.7%
• The policy is communicated to staff	14/15	93.3%
• The policy is visible to pregnant women, mothers and their families	12/15	80.0%
• The policy clearly states that the hospital protects and promotes breastfeeding, being an important health goal for mother, infant, family and society	15/15	100%
• The policy clearly states that health professionals implement hospital practices and apply clinical protocols recognized to promote and support breastfeeding	16/16	100%
• The policy openly states that it is responsibility of the Hospital Director to facilitate and support healthcare personnel in implementing hospital practices that promote breastfeeding	13/15	86.7%
• The implementation of the policy is periodically assessed	9/15	60.0%
• The policy requires training on breastfeeding of the staff	15/15	100%
• Training on breastfeeding is provided to staff within 12 months after entering the service	7//14	50.0%
• The policy regulates the exposure to pregnant women, mothers and their families of products promoting formula feeding	13/14	92.9%
• The policy states that at discharge from Maternity, milk formula must not be prescribed to mothers, who exclusively breastfeed	13/14	92.9%
• The policy states that at discharge from Maternity, clinical report must not include a pre-established space for the prescription of milk formulas to mothers, who exclusively breastfeed	12/14	85.7%

**Table 3:** Information on the exposure of the policy on breastfeeding in the facilities accessed by pregnant women, new mother and family. Data from hospitals with a policy

Answer options	Number of hospitals	Percentage
• Yes, the information on the policy on breastfeeding is provided only in Italian	6	40.0%
• Yes, the information on the policy on breastfeeding is provided also in the principal languages of the population who refers to our hospital	0	0.0%
• Yes, the information on the policy of breastfeeding is provided only in Italian; it is accessible also from the hospital website	2	13.3%
• Yes, the information on the policy on breastfeeding is provided also in the principal languages of the population who refers to our hospital; it is accessible also from the hospital website	4	26.7%
• No, the information on the policy on breastfeeding is not provided	3	20.0%
Total	15	100%

Policies were invariably developed by a multidisciplinary team including, in all cases, a neonatologist and/or a pediatrician. A nurse and/or a midwife were tipically part of the team, while the inclusion of a family representative and/or an anesthesiologist was less common (Table 4).

**Table 4:** Composition of the Working Group that developed the Policy on Breastfeeding. Data from Maternity Hospitals with a policy

	Number of hospitals	Percentage
• Pediatrician or Neonatologist	15/15	100%
• Obstetrician/Gynecologist	13/15	86.7%
• Anesthesiologist	3/13	23.1%
• Nurse	15/16	93.7%
• Midwife	13/14	92.9%
• Hospital Management Representative	13/15	86.7%
• Family Representative	6/15	40.0%

The policies consistently state that: (1) breastfeeding is considered an important health goal for mothers, children, the family and society, (2) hospital staff is committed to promoting breastfeeding and implementing postnatal practices such as skin-to-skin contact at birth and rooming-in to facilitate breastfeeding. Moreover, in 86.7% of the hospitals, the policy explicitly states that it is the responsibility of the Hospital Director to enable and support healthcare staff in implementing hospital practices that promote breastfeeding.

All policies indicate that maternity staff should be trained on breastfeeding; 50% of the hospitals with a breastfeeding policy require that health professionals receive training within 12 months of employment.

The policy states that the hospital protects families from inappropriate marketing of breast milk substitutes (86.7%) and prohibits the prescription of formula for exclusively breastfed newborns at discharge (92.9% of hospitals). Additionally, 80% of hospitals do not provide a preestablished box in the neonatal discharge summary for the routine prescription of milk formula.

Finally, a periodical assessment of the policy implementation is planned in 60.0% of the hospitals.

## Discussion

Our study shows that, at least in Italy, a mere 40.8% of hospitals with a NICU have established a breastfeeding policy for healthy newborns. This shortfall possibly reflects an underappreciation of the value of breastfeeding.

However, without information on the breastfeeding rates at discharge, it is challenging to compare the outcomes of hospitals with and without a dedicated breastfeeding policy.

It is well recognized that breastfeeding promotion deserves and requires the development of specific policies. Since the 1950s, when hospital childbirth became common, numerous cultural, organizational, and clinical barriers to the initiation of breastfeeding have emerged. Established breastfeeding hospital policies should be adhered to by health professionals to provide consistent and appropriate care irrespective of the individual provider. Written policies are considered a prerequisite for ensuring evidence-based care [6], which can increase the consistency of practices over time and helps organizational resiliency in case of emergencies.

For instance, the COVID-19 pandemic caused sudden, abrupt Maternity Hospital organizational changes that impacted the mother–infant relationship and breastfeeding initiation [8, 9]. Notably, the rate of exclusive breastfeeding at discharge was higher in Baby-Friendly Hospitals (BFHs) than in non-accredited centers for neonates born to mothers with COVID-19 infection at birth [5]. This difference may stem from the consolidated application of postnatal practices known to facilitate breastfeeding, such as skin-to-skin contact at birth and rooming-in [5]. Clearly, a consistent, written, evidence-based policy is essential for supporting these practices [6], although such a policy does not guarantee its appropriate implementation or effective communication to staff or that it will ultimately improves breastfeeding outcome.

Scientific societies and Health Authorities have provided recommendations for the aims, structure, and contents of an evidence-based breastfeeding policy for Maternity Hospitals [6, 10]. Nevertheless, there are few published studies assessing the extent to which breastfeeding policies are available, consistent and comprehensive [11].

Policies developed by BFHs can be presumed adequate, as they are examined during certification. The same cannot be assumed for policies developed by Maternity Hospitals that are not certified as Baby Friendly.

Only 26.7% of the breastfeeding policies were available in the main languages spoken by the population referring to the Maternity Hospital. Effective communication is crucial, as language barriers are associated with worse health outcomes. Thus, translating policies and educational materials for the target population is essential to deliver equitable health care [12].

A quarter of the Maternity Hospitals with a breastfeeding policy developed it in the last three years, possibly as a kind of reaction to the penalization of individualized care for mothers and newborn infants following the early stages of COVID-19 pandemic.

In Italian Maternity Hospitals with NICU, the working group responsible for developing the breastfeeding policy appears to have limitations in its multidisciplinary approach. The absence of a family representative may represent a missed opportunity to consider the needs of the family and perspective on postnatal care, according to the vision and the model of a “Family-Centered Maternity Care” [13, 14].

In the present study, all participating hospitals demand staff to receive training on breastfeeding. It is recognized that health professionals’ gap of knowledge on lactation, insufficient skills on breastfeeding management and inadequate attitudes negatively influence breastfeeding outcomes. Conversely, educational interventions have proven effective in enhancing knowledge, skills, and practices [15, 16].

Our study also focused on the prescription of infant formula at Maternity Hospital discharge. The policies appear to appropriately regulate formula prescriptions in most cases. It is well known that the infant food industry systematically targets health professionals offering sponsorship, incentives and training activities to build relationships and possibly influence health workers’ practices [17].

Despite the ongoing threat of inappropriate marketing, the number of countries with comprehensive legal measures to regulate such practices remains low. It’s important to note that Italian legislation only partially reflects the Code, and our survey was not designed to provide information on most requirements of the Code [7, 18].

Moreover, 60% of Italian Maternity Hospitals with a NICU do not conduct periodic assessments of policy implementation, which WHO and UNICEF recommend to ensure adherence to the policy [10]. Analyzing breastfeeding outcomes at discharge and measuring process indicators, such as the implementation of breastfeeding supportive practices are part of this periodic assessment.

To the best of our knowledge, this is the first study to explore breastfeeding policies among Italian Maternity Hospitals with NICU. Our survey also had broad geographic coverage, spanning 15 out of 20 Italian Regions. However, some limitations must be recognized.

Firstly, as our sample was restricted to Maternity Hospitals with a NICU, we cannot extrapolate our findings to the wider group of Maternity Hospitals without a NICU, which may have different levels of commitment to breastfeeding.

Secondly, the survey’s response rate raises concerns about the generalizability of our results. While a 40–50% response rate might be considered acceptable for survey-based research and can mitigate non-responder bias [19], there is a possibility that our findings overstate the commitment to breastfeeding promotion. This could lead to an optimistic view for Italy of the actual dissemination of breastfeeding policies in line with the Code.

## Conclusion

A limited number of hospitals in Italy have established a breastfeeding policy, and, when such a policy is in place, its quality appears to be suboptimal. This refers in particular to the lack of inclusion of family representative in the policy formulation working group, the insufficient translation of the breastfeeding policy into minority languages, and the absence of periodic assessment of policy implementation.

Consequently, Heath Authorities and Scientific Societies involved in perinatal care are called to intensify their efforts to foster the development, improvement and implementation of appropriate breastfeeding policies, as a crucial initial step in the promotion of breastfeeding.

### Electronic supplementary material

Below is the link to the electronic supplementary material.

Supplementary Material 1

## Data Availability

The datasets used during the current study are available from the corresponding author on reasonable request.

## Abbreviations

BFHI: Baby-Friendly Hospital Initiative
Com.A.SIN: Task Force on Breastfeeding of the Italian Society of Neonatology
COVID-19: COronaVIrus Disease 19
NICU: Neonatal Intensive Care Unit
SIN: Italian Society of Neonatology
SIP: Italian Society of Paediatrics
UNICEF: United Nations International Children’s Emergency Fund
WHO: World Health Organization

## References

[1] Victora CG, Bahl R, Barros AJ, França GV, Horton S, Krasevec J, Murch S, Sankar MJ, Walker N, Rollins NC.; Lancet Breastfeeding Series Group. Breastfeeding in the 21st century: epidemiology, mechanisms, and lifelong effect. Lancet. 2016;387(10017):475– 90.PMID: 26869575. 10.1016/S0140-6736(15)01024-7 10.1016/S0140-6736(15)01024-7 26869575

[2] Vaz JS, Maia MFS, Neves PAR, Santos TM, Vidaletti LP, Victora C (2021). Monitoring breastfeeding indicators in high-income countries: levels, trends and challenges. Matern Child Nutr.

[3] UNICEF/WHO. Baby-Friendly Hospital Initiative. Ten steps to successful breastfeeding from UNICEF and the World Health Organization.(Accessed May 24th, 2023). https://www.unicef.org/documents/baby-friendly-hospital-initiative

[4] World Health Organization. National Implementation of the Baby friendly Hospital Initiative 2017. WHO/NMH/NHD/17.4.

[5] Neo-COVID-19 Research Group: Marín, Gabriel MA, Domingo Goneche L, Cuadrado Pérez I, Reyne Vergeli M, Forti Buratti A, Royuela Vicente A et al. Authoring Group (Neo-COVID-19 Research Group). Baby Friendly Hospital Initiative Breastfeeding Outcomes in Mothers with COVID-19 Infection During the First Weeks of the Pandemic in Spain. J Hum Lact. 2021;37(4):639–648. 10.1177/08903344211039182 34374323

[6] Hernández-Aguilar MT, Bartick M, Schreck P, Harrel C.; Academy of Breastfeeding Medicine. ABM Clinical Protocol #7: Model Maternity Policy Supportive of Breastfeeding. Breastfeed Med. 2018;13(9):559–574.PMID: 30457366. 10.1089/bfm.2018.29110.mha 10.1089/bfm.2018.29110.mha 30457366

[7] World Health Organization. International Code of Marketing of Breast-milk Substitutes. Geneva: World Health Organization1981. Available from: https://apps.who.int/iris/bitstream/handle/10665/40382/9241541601.pdf?sequence=1&isAllowed=y

[8] Merewood A, Davanzo R, Haas-Kogan M, Giulia Vertecchi, Gizzi C, Mosca F, Burnham L, Moretti C (2022). Breastfeeding supportive practices in European hospitals during the COVID-19 pandemic. J Maternal-Fetal Neonatal Med.

[9] van Goudoever JB, Spatz DL, Hoban R, Dumitriu D, Gyamfi-Bannerman C, Berns M, McKechnie L, Davanzo R (2022). Updating clinical practices to promote and Protect Human Milk and breastfeeding in a COVID-19 era. Front Pediatr.

[10] World Health Organization (2018). United Nation Children’s Fund. Protecting, promoting, and supporting breastfeeding in facilities providing maternity and newborn services: the revised baby-friendly Hospital Initiative 2018: implementation guidance.

[11] Hawke BA, Dennison BA, Hisgen S (2013). Improving hospital breastfeeding policies in New York State: development of the model hospital breastfeeding policy. Breastfeed Med.

[12] Newell R, George S, Patel M, Dawe H, Roelas M, Wedmore F, Morris M, Rebecca Newell (2022). Improving the quality of translation for patients with language barriers. Eur J Public Health.

[13] Enkin MW (1973). Family centered maternity care. Can Fam Physician.

[14] Zwelling E, Phillips CR. Family-centered maternity care in the New Millennium: is it real or is it imagined? J Perinat Neonatal Nurs. December 2001;15(3):1–12. 10.1097/00005237-200112000-00002 11785574

[15] de Jesus PC, de Oliveira MI, Fonseca SC. Impact of health professional training in breastfeeding on their knowledge, skills, and hospital practices: a systematic review. J Pediatr (Rio J). 2016 Sep-Oct;92(5):436–50. Epub 2016 Feb 15. PMID: 26893208. 10.1016/j.jped.2015.09.008 26893208

[16] Balogun OO, Dagvadorj A, Yourkavitch J, da Silva Lopes K, Suto M, Takemoto Y, Mori R, Rayco-Solon P, Ota E (2017). Health Facility Staff training for improving breastfeeding outcome: a systematic review for step 2 of the Baby-Friendly Hospital Initiative. Breastfeed Med.

[17] World Health Organization. United Nation Children’s Fund 2022 How marketing of Formula milk influences our decisions on infant feeding. WHO https://www.who.int/publications/i/item/9789240044609. 10.1177/08903344221099943 35726490

[18] World Health Organization. The International Code of Marketing of Breast-milk Substitutes: Frequently Asked Questions (2017 Update), Geneva, Switzerland, WHO 2017. Licence: CC BY-NCSA 3.0 IGO. https://apps.who.int/iris/bitstream/handle/10665/254911/WHO-NMH-NHD-17.1-eng.pdf. (accessed Nov 16th, 2022).

[19] Story DA, Tait AR. Surv Res Anesthesiology. 2019;130(2):192–202. 10.1097/ALN.0000000000002436 30688782

