# Supplementary material for: Current hospital policies on breastfeeding: a survey from Italy
Source: Ital J Pediatr. 2024 Jan 25;50:21. doi: 10.1186/s13052-024-01581-5 (PMC10809559; doi:10.1186/s13052-024-01581-5)
Supplement: Supplementary file 1 — Supplementary Material 1 [file 13052_2024_1581_MOESM1_ESM.docx]

**2023 SURVEY on BREASTFEEDING POLICY**

**(Italian Society of Neonatology & Italian Society of Pediatrics)**

Please enter the following:

1° REGION OF THE HEALTHCARE FACILITY

2° CITY OF THE HEALTHCARE FACILITY

3° NAME OF THE HEALTHCARE FACILITY

4° FIRST NAME OF THE PERSON FILLING THE SURVEY

5° SURNAME OF THE PERSON FILLING THE SURVEY

6° HEALTHCARE FACILITY RELEVANT CHARACTERISTICS

- Neonatal Care Unit
- Neonatal Care Unit and Neonatal Intensive Care Unit (NICU)
- Neonatal Care Unit & Paediatric Unit

7° IS THERE IN PLACE A POLICY FOR BREASTFEEDING?

- Yes, we are a certified Baby Friendly Hospital and we have a policy in place that fully endorses the WHO International Code of Marketing of Breastmilk Substitutes (“the Code”)
- Yes, we are on track to become a Baby Friendly Hospital and we have a policy in place that fully endorses and support the WHO International Code of Marketing of Breastmilk Substitutes (“the Code”)
- Yes, we do have a policy in place, although it does not fully endorse the Code
- No, we don’t have any policy in place, but we are in the process of developing one
- No, we don’t have any policy in place.

Note: any NO answers will determine the end of the survey, any YES answer will allow to continue with the survey

8° PLEASE UPLOAD THE POLICY HERE

9° WAS THE POLICY DEVELOPED BY A MULTIDISCIPLINARY GROUP?

- Yes
- No

10° PLEASE CHECK THE CORRESPONDING BOX FOR EACH PROFESSIONAL INCLUDED IN THE MULTIDISCIPLINARY GROUP

- Paediatrician or neonatologist
- OB/GYN specialist
- Anesthesiologist
- Nurse
- Midwife
- One component from the Department of Health Management of the Hospital
- Representative member of the families association
- Other:

11° WHEN WAS THE POLICY APPROVED?

- Before December 31st, 2019
- From January 1st, 2020

12° WAS THE POLICY ADEQUATELY COMMUNICATED TO ALL THE STAFF INVOLVED IN CHILD AND MATERNAL CARE?

- YES
- NO

13° HAS THE POLICY BEEN ATTACHED IN THE HOSPITAL AREAS WHERE MATERNAL AND CHILD CARE ARE BEING OFFERED TO PREGNANT WOMEN, MOTHERS AND FAMILIES?

- YES, ALTHOUGH ONLY AVAILABLE IN ITALIAN.
- YES , IN THE MOST PREVALENT LANGUAGES SPOKEN BY THE COMMUNITY.
- YES, ALTHOUGH ONLY IN ITALIAN. HOWEVER, IT IS ALSO VISIBLE ON THE HOSPITAL WEBSITE.
- YES , IN THE MOST PREVALENT LANGUAGES SPOKEN BY THE COMMUNITY AND IT IS ALSO VISIBLE ON THE HOSPITAL WEBSITE.
- NO

14° DOES THE POLICY CLEARLY STATE THAT THE HOSPITAL DIRECTION PROMOTES BREASTFEEDING AS AN IMPORTANT HEALTH GOAL FOR MOTHERS, BABIES, FAMILIES AND SOCIETY?

- YES
- NO

15° DOES THE POLICY CLEARLY STATES THAT THE HOSPITAL DIRECTION SUPPORTS STAFF TO IMPLEMENT ALL PRACTICES AIMED AT PROMOTING BREASTFEEDING, SUCH AS THE ROOMING IN AND THE SKIN-TO-SKIN PRACTICES?

- YES
- NO

16° DOES THE POLICY CLEARLY STATES THAT ALL STAFF IS COMMITTED TO PROMOTING BREASTFEEDING, THROUGH THE IMPLEMENTATION OF PRACTICES ENABLING PRACTICES SUCH AS SKIN TO SKIN AND ROOMING IN PRACTICES, AND THROUGH THE APPLICATION OF ADEQUATE PROTOCOLS ON BREASTFEEDING?

- YES
- NO

17° IS THE POLICY IMPLEMENTATION PERIODICALLY ASSESSED?

- YES
- NO

18° PLEASE STATE IN FEW LINES HOW IS THE POLICY IMPLEMENTATION MONITORED:

19° IS IT MANDATORY FOR ALL STAFF TO UNDERGO BREASTFEEDING TRAINING ACCORDING TO THE POLICY?

- YES
- NO

20° IS THERE A SPECIFIC TIMEFRAME FOR WHEN TRAINING SHOULD BEGIN AFTER EMPLOYMENT ?

- LESS THAN 12 MONTHS AFTER EMPLOYMENT
- MORE THAN 12 MONTHS AFTER EMPLOYMENT
- I DO NOT KNOW

21° DOES THE POLICY PROVIDE PROTECTION FOR FAMILIES AGAINST MARKETING PRACTICES PROMOTING ARTIFICIAL MILK?

- YES
- NO

22° DOES THE POLICY SUPPORT WITHOLDING THE PRESCRIPTION OF FORMULA MILK WHEN A BABY IS DISCHARGED FROM THE HOSPITAL EXCLUSIVELY BREASTFED?

- YES
- NO

23°Does the policy clearly state to remove the section in discharge documents that recommends formula milk as a routine prescription?

- YES
- NO
